# Abstracts and Gleanings

**Published:** 1880-06-20

**Authors:** 


### ABSTRACTS AND GLEANINGS.

**The Active and Passive Inhalation of the Nascent Chloride of Ammonium in Acute Affections of the Respiratory Tract.**—Ephriam Cutter, M.D., of Boston (Virginia Med. Monthly), writes as follows upon this subject:

When the vapors of ammonia and muriatic acid are brought together, immediately at the points of contact a dense cloud is formed that has the microscopic properties of smoke. Under the microscope of 400 diameters, I find that this cloud is made up of feathery crystals that resemble the lighter crystals of snow, and, from their many surfaces, appear white like snow, milk or fine sand. Four needles cross each other at their centres at angles of 45 degrees; so that this smoke is made up of an immense number of minute microscopic crystals that freely float in the air in all directions. This physical property is something wonderful. We are not amazed at the formation and floating in the air of snow crystals, as the atmosphere holds watery vapor in solution; nor are we surprised at the formation of the crystals from the ammonia and muriatic acid vapors; but that crystals of the specific gravity of 1.45 should not at once settle to the ground instead of diffusing themselves as the incomplete products of combustion, we call smoke, is in my mind worthy of remark.

Now, though the chloride of ammonium (*alias* sal ammoniac) is mentioned in all pharmacopœias and in Sancscrit, still, save among the Germans, and latterly among ourselves, no advantage has been taken of this property of aerial diffusion so as to administer it by inhalation. No mention is made of this use in Wood and Bache. But the Germans, Kirkwood and some unprofessional people in this country have given it in chronic affections of the air passages. It is easy to see that the crystals inhaled will impinge in substance on the mucous membrane of the respiratory tract, and serve as if it had been made a topical application; and this, too, in the mildest mode of direct application, having no medium or vehicle of communication save the atmospheric air.

What is meant by "nascent?" This means "born," or, as new birth is the beginning of existence, the Germans have applied the term to the newly-formed chloride of ammonium. They seem to think that there is an especial advantage in having the salt as fresh as possible, as, if it is used just as it is formed, its therapeutical power is greater. Be this as it may, the salt does not long remain in the air, but settles down or is blown off, or diffused in the air. The chemical union is expressed by a simple formula. $NH_4O$ signifies ammonium; HCl signifies muriatic acid; $NH_4Cl$ signifies chloride of ammonium; HO signifies water. ($NH_4O$—HCl=$NH_4Cl$—HO.)

What is meant by active and passive inhalation? *Active* is where the patient draws in the nascent chloride, as a smoker uses a pipe or a cigar. *Passive* is where the apartment is filled with the crystals, and the patient inhales as a non-smoker does in a car full of smokers; or the crystals may be blown into the face of the patient. The passive use is indicated when patients are unable or refuse to actively inhale—as in cases of very sick people, children and infants. This feature of passive inhalation, and the use of the nascent chloride in acute affections, characterize this contribution to the therapeutics, the nomenclature and the apparatus of this subject.

The writer here gives illustrations of apparatus for inhaling the nascent chloride, and adds:

To those who are so situated as to desire to make their own instruments, I may say that useful ones have been made by me from empty pickle jars of two quarts capacity—boring holes through the cork with rat-tailed files, cutting out a V-shaped segment from the cork at its periphery; take a hairpin, impale the sponge on the loop and under the cork, bending a glass tube over a flame, or using a small lead tube (one may be made by rolling up a sheet of lead on a cylinder like a common lead pencil). Often I have used a common gimlet screw to make the holes in the cork—burying the screw in the cork, then forcibly withdraw it, and repeat till the hole is made; then, using the threads of the screw as a rasp, enlarge the hole to a proper size. In lieu of a sponge, a piece of common cotton cloth impaled on the hairpin has answered well.

There are many nascent chloride apparatuses in the market, but they are all for active inhalation as far as I have seen. Moreover, the tubes are too small. I have thought that they should be larger than tracheotomy tubes for viability and to avoid friction; also, the expense is too great in my opinion. While I am not prepared to say that my own apparatus is the best, I can testify that it has worked to my own satisfaction, especially when the patients were too weak to inhale through a tube, or could not swallow their medicines, or where they were sensitive children or helpless babes. (See Boston Journal of Chemistry, February, 1874).

EXAMPLES FOR USING.—*Rhizopod Colds.*—Some readers may recall allusions that I have made in this journal to cases of this kind. It is astonishing how prompt the relief is sometimes; for example, in croupal symptoms associated with rhizopods.

In December, 1870, my son, aged 4½ years, contracted the rhizopod cold. The family had had it previously. I found the living forms of the asthmatos in the nasal secretions. In the day time, he would be comparatively well, and at night on going to bed, though a little restless slept well. But after the family had retired and got quietly settled down into primary sleep, we were soon roused by the typical croupal cough that has so often alarmed parents and startled physicians. From laryngoscopical examination, I am satisfied that the physical cause of the brassy clangor in the cases explored was from the thickened reddened infiltration of the soft areolar submucous tissue about the false vocal cords. In the present case, I could not get a view of the larynx proper any farther than the epiglottis, but inferred from the contagious nature of the cold—from the live rhizopods found—that the irritation caused an inflamed condition of the peripheral part of the larynx so that he could produce at will cough clangor. Anyhow, the passive inhalation of the nascent chloride was followed by the immediate arrest of the cough and continuous sleep through the rest of the night. My wife insists that this change of treatment (for which we thank Dr. Salisbury)is a great improvement upon our former methods, inasmuch as formerly, with like symptoms, a sickness of several days' duration was sure to ensue—to say nothing of the mental wear and tear occasioned by witnessing and expecting dyspnœa of one's own children. In the morning, on examining the nasal excretions, forms of the rhizopods were found, but they were all dead.

*Remarks*—A few pinches of powdered sulphur burnt in the apartment will relieve just as quickly as the nascent chloride. Moreover, those physicians who are not familiar with the use of the microscope, may, I think, diagnosticate the influenzas depending upon the rhizopods by the results of inhaling these remedies, according to my own observations. The same is true of the asthma caused by these parasites. I have seen whooping-cough also cured by them.

*Typhoid Pneumonia* is a very dangerous disease in my experience. The first use of the nascent chloride in this disease, as far as I know, was in the case of a strong Irish-American farm-hand, 22 years old, who in a midwinter of protracted and low temperature, went off one evening and got drunk. Instead of seeking his bed, on his return, he turned in on some hay mown in the barn. The atmospheric temperature was, on an average, 15 degrees below zero. Thus he contracted a severe cold that resulted in typhoid pneumonia—that is, inflammation of the lungs with typhoid symptoms. When I saw him, he was in a third-story chamber of the farm-house, too sick to move; water in a tumbler near the bed was frozen one-half inch thick, and there was no means of making a fire. Fever somewhat high; pulse quick and weak; copious glairy, rusty sputa; physical signs of hepatization of the right lung, lower lobe. Ordinary treatment was administered. Soon his throat became so sore that he could not swallow. He even had muttering delirium at times. Respiration difficult and quick. He took but little notice of anything. His lungs were filled with secretions. Sweating copiously; weakness. Sputa darker in color. Urine high colored; some diarrhea. Here was a peculiar situation—no medication possible by the stomach. So I was forced to resort to inhalation, as it was the only possible method of interference. A nascent chloride of ammonium was extemporized. The man was roused, and fortunately made to understand what he was to do. He actively inhaled ad libitum. Never did anything seem to work so well. There was an immediate improvement in every respect. Fortunately, a thaw came on. He was successfully moved into better quarters. His recovery was prompt and complete. The nascent chloride was the chief medicine used after it was begun with.

*Remarks.*—Doubters are apt to meet such statements as these by doubting everything. While I am not prepared to say that this man might have gotten well without any treatment, still I do say that the nascent chloride of ammonium was thoroughly administered; and taking this case, with others like it, I base on this evidence the assertion that I think I have a right to say that he would have died without this relief; and it was a relief. The patient said so. He kept asking for the apparatus, and inhaled most of the time. Had he been too weak to inhale, the passive mode would have been adopted. Others have had like experiences.

*Capillary Bronchitis.*—Some ten years ago a woman, 67 years old, had been sick with cough and cold for about six months. She had good medical attendance, but with no relief. The cough was persistent; sputa copious, white and glairy; both sides of chest clear on percussion; full of sonorous and sibilant crackling inspiratory and expiratory rales; loss of flesh and strength; blood showed no physical signs of phthisis or syphilis. Immediate relief followed the use of the nascent chloride of ammonium in this case. In one week's time the cough and rales had well nigh disappeared, and health was restored. She has had no return of the affection.

This is not a complaint that ordinarily gets well so quick; indeed, it is apt to baffle the most skillful following up. I have seen the nascent chloride give so much relief in these affections that I do not feel that I make a mistake when I attribute the relief to the means employed.

*The Bronchitis of Children and Infants.*—I wish to speak a word in behalf of these delicate subjects. All know how difficult it is to treat this class of cases, and that homeopathy has its supposed success in them, as it is so easy to give these patients medicines that taste like sugar. But it seems to me that Hahnemann never ordered a medicine so easy to take as chloride of ammonium. The child cannot help inhaling, and often reaches out its arms for the inhaler, showing that the inhalation must be pleasant, while at the same time we know that a common sense and appreciable influence must be exerted by the crystals. But putting this aside, I have seen cases where the favorable effects were marked and satisfactory.

The inhalation of the nascent chloride is one of the best modes to relieve or break up a cold. Generally, those who use the apparatus are glad to have it on hand for domestic use.

In conclusion, I hope that I have not given the impression that this remedy is "a cure-all;" it is not. Still I think that its use should be delegated to laymen, as I believe that, if the profession have the same experience with it in the acute affections named, that many lives will be saved and some severe sicknesses will be mitigated and made pleasant.

**Advances in Materia Medica.**—Within the last year or two, the contributions to the list of materia medica have been large. Among these novelties, a few possess valuable therapeutic properties; while the greater portion are mere novelties or old remedies revived, which will be classed as worthless as soon as they cease to pay the unscrupulous advertiser who recommends them to the over-credulous of our profession and to the public, by the most exaggerated statements of their wonderful virtues. To burden this paper with an enumeration of them, in my judgment, would be foreign to my subject. True advance is shown only after the agents suggested have been duly investigated by trustworthy and intelligent seekers after truth, and have been found efficacious in disease. Of such, I will briefly allude to.

*Araroba*, or goa powder—a Brazilian product, which came to the attention of the medical world through its efficacy in the Portuguese colonies, in Africa and Asia—places noted for the inveterate character of many loathsome skin diseases—as a remedy particularly for psoriasis. It has been examined chemically by Prof. Attfield. He discovered its active principle to be crysophanic acid. The crude powder, as well as its active constituent, are now well endorsed as valuable medicinal agents, and the range of its usefulness much extended.

It is interesting to observe that our common yellow dock—rumex—that has enjoyed a certain amount of reputation among domestic remedies as a remedy for cutaneous diseases, such as ringworm, itch, etc., is found to be rich in this same crysophanic acid. A hint is here given to our country practitioners to utilize this indigenous plant in their practice.

*Thymol*—obtained from oil of thyme—has become a popular antiseptic and disinfectant. Perhaps no greater praise has been awarded to it than that arising from the fact that it is one of the few new remedies that meets with approbation in the pages of the *National Dispensatory*, last edition. As a remedy, it has the advantages over carbolic acid in its not being irritating or corrosive to the tissues of the body, and in possessing an agreable odor; and it is said to be not inferior to it in its disinfecting property. These advantages entitle it to a place in our materia medica. An ointment of it (30 grains to the ounce) is recommended as a substitute for the obnoxious tar ointment, in all cases where the latter may be efficacious.

*Ethylates of Sodium and Potassium*, recommended by Dr. Benj. W. Richardson, of London, appear to be valuable caustic agents. In the year 1870, Dr. Richardson, at a meeting of the Medical Society of London, read a paper on this subject; and again in the London Lancet (American edition) July, 1879, he calls the subject to the notice of the wider circle of medical practitioners.

*Sodium Alcohol*, or *Sodium Ethylate*, is prepared by simply treating absolute alcohol with metallic sodium. As thus obtained, it is in the form of a thick, white product. It is a most convenient caustic. Laid on dry parts of the body, it is comparatively inert; but so soon as the parts to which it may be applied give up a little moisture, its caustic action can be observed. Its action may be developed so gradually as to be hardly perceptible, or increased, by the intelligent use of it, to the most potent caustic effect.

The sodium salt is recommended more particularly for general use from its being a milder caustic than the potassium salt. For practical use, it is recommended for destroying morbid growths that are not favorable for excision by the knife. It may be used either externally or by injection into the morbid tissues. When the pain is felt to too great a degree, or when its caustic action is too pronounced, it may be quickly checked by a few drops of chloroform, which decompose it into an inert chloride salt and an ether.

Dr. Richardson has used these ethylates repeatedly in his practice, with much success, and recommends them chiefly in three forms of disease, viz.: Cutaneous nævus, lupus, and malignant ulcer. The remarks of Dr. Lauder Brunton, before the Medical Society of London, and published in the Lancet, January, 1879, are very interesting in the report of several cases of nævi, treated most satisfactorily with this remedy.

(The following notes on the two new *myotics*, eserine and pilocarpin, and on the new *mydriatic*, duboisine, are kindly contributed by Dr. Joseph A. White, of Richmond.)

*Eserine* is one of the alkaloids of the calabar bean (physostigma venenosum), and was first introduced into ophthalmic practice by Prof. Lagueur, of Strasbourg, in 1875. It is most commonly used in the form of a neutral salt—the *sulphate of eserine*. A one per cent. solution (about four grs. to the ounce of water), if dropped into the eye, produces contraction of the pupil, spasms of the ciliary muscle, shortening of the range of vision, and a decrease of the radius of corneal curvature. It is, therefore a *myotic*, and its local effects are antagonistic to atropine and duboisine. Although it sometimes irritates the conjunctiva, I do not think it ever produces any constitutional symptoms.

As a local application, it has been used in corneal ulceration, more especially in the peripheral forms with tendency to perforation; in abscess of the cornea; in corneal suppuration following cataract extraction; in keratócomus; in commencing sympathetic irritation; in paresis of the accommodation, and in some forms of glaucoma with very favorable results.

In keratitis, it has not developed any specific action, and in glaucoma has disappointed the expectations that were at first formed of it as a curative means in this affection. In the acute form, it has, in some few cases, seemed to arrest the disease; in the sub-acute form, it is a palliative measure that may be resorted to as preparatory to iridectomy; but in neither form can it take the place of this operation. In chronic glaucoma, it may be positively dangerous, because of a tendency it has shown of bringing on acute attacks.

*Pilocarpin* is the active principle of jaborandi (policarpus penatifolius) which was discovered by Mr. Hardy, and introduced into ophthalmic practice. Its two salts—the muriate and nitrate—are both used, but more especially the former. Its local application causes contraction of the pupil, and increased secretion of the lachrymal gland, and by absorption may produce some of its constitutional effects. These are profuse perspiration, ptyalism and slight nausea, and in larger doses, vomiting, pain in abdomen and genitals, with general prostration.

As, when administered internally or hypodermically in such doses as do not produce the violent physiological effects, it improves the peripheral circulation, decreases the temperature of the body, and causes vascular depletion of the eye, it is a valuable remedy in treating iridocyclitis, in choroiditis exudation, in opacities of the vitreous, in retinal detachment, in glaucoma and in commencing atrophy of the optic nerves. In fact, some claim that it is almost a specific against commencing atrophy.

Locally, it is used in rheumatic and serous iritis, and in specific and rheumatic keratitis. As a myotic, it is inferior to eserine, as the latter produces no unpleasant effects beyond occasionally some irritation of the conjunctiva. For local application, a one per cent. solution (about 4 grs. to the ounce of water) of the muriate of pilocarpin is most commonly employed. Hypodermically, I have administered it in doses varying from gr. 1/6 to gr. 1/2 daily, according to the susceptibility of the individual to its influence, always regulating its administration so as to stop short of nausea and vomiting, as the perspiration, ptyalism and reduction of temperature are sufficient manifestations of its physiological action. In some patients, I have seen it followed by profuse perspiration without ptyalism, and *vice versa*.

*Duboisine*, the latest remedy in ophthalmic therapeutics, is the active principle of the duboisia myoporoides, an Australian plant of the family of Scrofuloricees. The alkaloid was first obtained from the plant by Gerhard, of London.

Its action is very similar to atropine, but more powerful. It therefore belongs to the class of mydriatics, and is antagonistic to eserine and pilocarpin.

It is used in the same class of cases as atropine, but is superior to the latter, inasmuch as it dilates the pupils, and paralyzes the accommodation more promptly; its effects are less lasting, and it does not irritate the conjunctiva. From this latter property, it is indicated in cases which show an idiosyncrasy against atropine.

It also has the power of dilating the retinal veins; and further investigation of this property may show it to be a very valuable remedy in some affections of the fundus oculi. In rare instances (as reported by Dr. Seeley, of Cincinnati), when a strong solution has been used too continuously, it has produced vertigo and drowsiness.

The strength of the solution for local use is from two to four grains to the ounce.

All these remedies are very high-priced, and have not yet come into common use; and although many experiments have been made as to their applicability, their exact value as therapeutic agents has not yet been determined.—*Dr. Robinson, in Va. Transactions.*

**Treatment of Diseases of the Tonsils.**—Dr. George M. Lefferts read a paper including this subject. In the discussion, Dr. J. H. Douglass said that one reason for the chronic condition of enlarged tonsils was, in his opinion, a partial constriction of the tonsils by the anterior and posterior pillars of the fauces. The constriction resulted from a catarrhal inflammation of the tissues. A point in treatment was, to reach the posterior arch; and to effect this he found that the nozzle of syringe could be carried far enough back to wash out any concretions of mucus and apply appropriate remedies. That congestion could increase the size of the tonsil to three or four times its natural size he had proved by making post-mortem injections with lard. He had seen many tonsils, both large and small, removed, but he was not so favorably impressed with the operation as he had been. In enlarged tonsils he introduced the nozzle of a syringe into the crypts and washed them out. He had found that by this method of treatment, together with attention to the posterior arch, the tonsils soon became reduced in size. In regard to the hemorrhage after the operation, he had seen a number of cases in the practice of his brother-in-law, the late Dr. Horace Green, and had found that clots forming in the veins were the cause in many cases. After these clots were washed out the bleeding ceased.

Dr. Beverley Robinson was accustomed to apply to the crypts of the enlarged tonsil a probe coated with nitrate of silver. Many patients had a prejudice against the removal of the tonsils.

Dr. Roosa was sorry that the name "throat-deafness" had come into use. It was unscientific, and should be replaced by the term catarrhal deafness. He did not believe that the tonsil pressed on the Eustachian tube, for in quinsy there was not, as a rule, deafness during the attack. He believed that the deafness was due to coincident catarh of the Eustachian tube.

Dr. R. P. Lincoln had known pain in the ear to accompany inflammation of the tonsils, and he was induced to believe that it was due to a nervous cause, inasmuch as branches of the glosso-pharyngeal and the otic ganglion were supplied to the ear. He had recently removed a mass from the tonsil of a patient who suffered from pain in the ear without disease of the ear. Relief followed the removal. He was in the habit of applying to the crypts of the hypertrophied tonsil a solution consisting of one part of carbolic acid and eight parts of the compound tincture of iodine. Cases usually improved after three or four applications. A Cuban lady came under his observation, suffering, as we supposed, from malignant disease of the tonsil. A mass of the size of a walnut, was removed and there followed complete relief. The pain was very severe, and over the parietal region of the affected side the hair was blanched over a space as large as a silver dollar.

Dr. Frank H. Bosworth thought that some cases of acute tonsillitis may be aborted by giving ten grains of quinine and subsequently ten drops of Fleming's tincture of aconite every hour until the physiological effects were complained of. He was of the opinion that nightmare in children was often the result of tonsillitis.—*Dr. Barker, in N. Y. Acad. of Med., N. Y. Med. Journal.*

**A Case of Typhoid Fever Treated with Carbolic Acid Internally.**—Dr. Henry Weekes, in a letter to editor of London Lancet, says :

If you will oblige me by bringing before your readers the following case, it may induce those who have more opportunities of meeting with typhoid fever than I have to see if similar results may be obtained in a more extensive trial.

On January 24th, I visited Ellen S——, aged eighteen, living in a detached cottage in the country. She had taken to her bed two days before, having been previously ailing for a week. I found her in a very febrile condition, with highly flushed face, anxious expression, tongue furred, and bowels relaxed three or four times a day; very little headache. Pulse 108; temperature (11 a.m.) 102–5°; skin dry. With the usual instructions as to diet, etc., I prescribed acetate of liquor ammoniæ and compound tincture of camphor.

Jan. 25th.—No apparent change. She complained much of sleeplessness. Ordered compound ipecacuanha powder and mercury with chalk at bed time.

26th. Some sleep. Tongue browner; diarrhœa lessened; some spots on abdomen, but scarcely observable. Pulse 109; temperature (12 a.m.) 103°.

27th. Much flushed; tongue more glazed; pulse 108; temperature 103–5° I prescribed glycerine of carbolic acid, six minims, every four hours.

28th.—Face less flushed; tongue moister, but spots more distinctly colored; pulse 100; temperature 101°. Continued carbolic acid.

29th.—Patient cheerful after a good night; diarrhœa nearly ceased; tongue rapidly cleaning; spots less evident; pulse 89; temperature 99°.

From this day she made a rapid recovery, sat up on February 1st, and was down stairs on the 4th.

Now, the sudden subsidence of fever on the administration of carbolic acid may be merely a coincidence, and the same remark may be made regarding the supposed results of other medicines in solitary cases; but such remarkable coincidences are certainly suggestive of more extended experiments. Until disproved, it appears to me more probable than otherwise that one was the effect of the other. Our great authority, Sir W. Jenner, says: "I have never known a case of typhoid fever cut short by any remedial agent—that is, cured. The poison which produces any one of the acute specific diseases (to which order typhoid as much as small pox belongs) having entered the system, all the stages of the disease must, as far as we know, be passed through before the recipient of the poison can be well."

Yet I submit that this very disease (small pox), here linked with typhoid fever, is undoubtedly modified and cut short in its course by introducing vaccine lymph into the system, even when vaccination has been performed so late as to run its course concurrently with the small pox. Why then should we remain content with thinking that typhoid fever must continue to the end unchecked?

**The Cure of Cancer.**—Professor John Clay, of Birmingham, has published some remarkable cases of cancer of the uterus cured by the internal administration of Chian turpentine. We give the following as one of the best marked cases. The patient (æt. 32) came to the Queen's Hospital after having been discharged incurable at the Women's Hospital. She was greatly depressed. She had had repeated floodings, and suffered greatly from pain during the last five months. Constipation very troublesome, probably due to opiates. She was found to be suffering from epithelial cancer of the os and cervix uteri, but not involving the vagina. There was a cancerous mass of the posterior parts of the os and cervix of the size of a goose egg. This growth pushed the os uteri towards the pubis, almost preventing that part from being felt.

A mixture containing six grain doses of Chian turpentine dissolved in ether and suspended in mucilage was taken three times a day, and from this period a very rapid diminution of the growth took place, so that by the sixteenth day it had almost entirely disappeared. The os uteri was now in situ, admitted the finger readily, and the vessels of the tumor assumed a shrivelled appearance. A solution of perchloride of iron was then used daily with excellent effect.

In the ninth week the patient suffered from spasmodic pains in the back and abdomen, which was attributed to the medicine. Iodide of calcium was then given for a fortnight. After this Chian turpentine was resumed and an arsenical lotion was used locally.

Under this treatment the woman very rapidly improved, the pains ceased and the parts become much reduced in size and more moveable. She was sent to a sanitarium and discharged convalescent.

Professor Clay says the Chian turpentine seems to act on the periphery of the growth with great vigor, causing the speedy disappearance of cancerous infiltration, and thereby arresting the further development of the tumor. It appears to dissolve all the cancer cells. It is a most efficient anodyne causing an entire cessation of pain in a few days. The Professor, whose name is a sufficient guarantee for the diagnosis and the results of treatment, does not affirm that Chian turpentine is a positive cure for advanced cancer of the uterus. Nevertheless all the patients treated are still living, their disease has been arrested and has all but disappeared, and it certainly relieves the pain in a manner which cannot be said of any other remedy.—*London Lancet.*

**Purgatives--For what Objects shall they be Administered in Treating Cases of Diarrhea and Dysentery.**—These are given by Dr. J. J. Woodward (Part II, History War of Rebellion, page 726) thus: (1) To evacuate noxious matters contained in the alimentary canal. All admit that undigested food and substances undergoing putrefactive changes should be removed from the intestines of persons suffering from fluxes. To meet this object any pleasant, efficient purgative would suffice. (2) To increase or modify the secretion of the intestinal mucous membrane. It seems conclusively shown by Breeger that the neutral salts certainly possess the power of producing a discharge of watery fluid from the intestinal mucous membrane, mingled with increased secretion. Thus it seems that neutral salts produce greater increase in the intestinal secretions, with less irritation, than can be obtained with any other purgatives with which we are acquainted. It may reasonably be expected that, especially in the early stages of a catarrhal inflammation of the intestinal mucous membrane, such as occurs in acute catarrhal diarrhea and dysentery and in the catarrhal stage of diphtheritic dysentery, the action of purgatives which decidedly increase the intestinal secretion will relieve the congestion of the intestinal blood-vessels, and that, after the temporary increase of vascularity produced by their action has subsided, an improvement in the condition of the mucous membrane will follow. In chronic conditions moderate catharsis occasionally is beneficial in washing out the contents of the alimentary canal. (3) To increase the biliary secretion in alcoholic conditions, Rutherford has shown that a number of cathartics will increase the biliary secretion. The local irritation produced by some of these render them unsuitable for use in intestinal fluxes. Others are so mild as to readily fulfill the proposed indication. Of these are sulphate and phosphate of soda, Rochelle salts and rhubarb; these are cathartics upon which we should rely in the treatment of fluxes complicated with deficient hepatic secretion, instead of invoking the doubtful properties of calomel.—*Detroit Lancet.*

**When to Perform Ovariotomy.**—Edward Borck, M. D., of St. Louis, Mo., in an article on ovarian tumors, published in the Obstetric Gazette for March, 1880, gives it as his opinion that it would be better to recommend the operation rather a little too soon than too late, and that the early operation will be the accepted rule in the future for the following reasons:

1. That abdominal section is not by far so dangerous under the antiseptic method, as prior to this, without it. Peritonitis is thereby claimed to be prevented, and we are informed by good authority that we now can operate at least one year sooner. Observe the success Schrœder and others had since they adopted the antiseptic method. (See also Nathan Bozeman's remarks on ovariotomy, New York Medical Record, July and August, 1878).

2. As the peritoneal cavity has been opened and exposed in other operations, without peritonitis following, and where waiting for distention was out of the question. For Dr. Martin, of Berlin, has removed five times a floating kidney, four times successfully, by abdominal section. Marion Sims tells us that his operations before Listerism would have been wholly unjustifiable.

So the danger of traumatic peritonitis is greatly reduced by Listerism, and the argument that in anemic patients the danger of secondary hemorrhage is not so likely to occur, seems to me not very solid, though it is true where there is no blood none can flow. I myself should prefer rather a little too much blood than too little; too much we can easily reduce, and we can control a too rapid flow of blood by contracting the blood-vessels by ergot, and may thereby prevent oozing. But where there is too little, more is hard to produce.

Six times vaginal ovariotomy has been performed in this country, all early operations, and successfully. The first, I believe, by T. G. Thomas, though it is not his belief that tho scope of this plan will ever be very great. But I myself believe a good deal speaks in its favor.

In conclusion, I would say that the object of this communication is to call attention to the above arguments, and especially to the inadvisability recommended waiting or delaying the operation.—*Medical and Surgical Reporter.*

**The Dissemination of Typhoid Fever.**—Dr. Robert Volz, Grossherz. Bad., Obermedizinalrath and Bezinesarzt, at Carlsruhe, publishes the results of his investigations concerning the spread of typhoid fever as deduced from the examinations from official sources of sixty-two epidemics. The material included both the large and small epidemics as they prevailed in various parts of the grand duchy of Baden, since July, 1871. The number of sick amounted to 3,300, with 400 deaths.

The author's report shows that typhoid fever is brought into a house by a patient sick with the disease, and then disseminates itself through the household. It can be spread by intercourse with the patients or stay in the house of visitors. The report does not give exact information as to whether a visitor may himself remain free but carry the disease to others in other houses. But the report does prove that a single patient may start an epidemic in a place entirely free of the disease hitherto.

Typhoid fever is thus infectious from the sick to the well—that is, it is contagious. But infection is only possible when its specific poison adheres to a material substance by which its reproduction may be effected.

It is certain that the typhoid germ may spread itself from the washing. And drinking water may spread the disease, if the drinking water contain the germ.

As preventives of the spread of the disease, the following rules are given: The physician must give public notice of every case of typhoid fever. So soon as the diagnosis is established, the patient must be isolated as much as possible. The house or room in which the patient lies should have posted on it a caution notice. Parties must not be removed to a house free of the disease, except to a hospital. The washing must be done by itself.

The time of the year has a distinct influence on outbreaks of typhoid fever. The greatest maximum is in the fall, and the highest mortality is in November. There is no mention of the influence of subsoil water.—*Med. Neuigk. f. prakt. Aerzte.*

**The Tongue.**—We shall not attempt to describe all the appearances, as found in various diseases, but only call attention to the following:

1. The broad, pallid, white-furred tongue, whether found in acute or chronic disease, and even in the same disease at different stages, it matters not, always indicates the want of the alkaline elements of the body. The patient that has such a tongue never wants an acid drink, because his system is already acid, whether the acid is in the stomach or blood, it matters not. In such cases, with our usual prescription, we must give alkalies, such as sulphate or bicarbonate of soda, in order to neutralize the acid. Then, and not till then, will our remedies act kindly and much more promptly, and the patient improve more rapidly. Now, if acids are prescribed when the tongue is broad and pallid, the system being full of acids already, it will be seen at once that no improvement could or should be expected. I remember, before I was aware of this rule, that I could not see why my remedies did not improve the patient; but now I give the proper alkali, removing the acid; then the remedies act more promptly, the tongue changes to a better appearance, and the patient improves correspondingly. This acid condition of the system is probably more often found in acute than in chronic diseases; at least, that is my observation. It needs but a trial to test this principle; have never known it to fail. I do not pretend to explain why the tongue looks thus when there is a superabundance of acid in the system.

2. The deep-red, sleek tongue, with a slight coat at the base, indicates just the opposite condition; here the system is in an alkaline condition, no matter in what form of disease. Then some kinds of acids must be given, to neutralize the superabundance of the alkali, before other remedial agents will, or even can, have a good effect. All persons that have this peculiar tongue desire acid drinks. This tongue is often (though not always) seen in erysipelas, typhoid fever, and many other diseases where the tincture of chloride of iron or muriatic acid will be the leading remedies. But if the system was in an acid condition, known by the broad, pallid tongue, those remedies would do more harm than good. Now I do not wish to inculcate the idea that alkalies or acids will cure any disease, but the above appearances of the tongue are indications of specific physical conditions, and those remedies are adjuvants, paving the way and assisting specific remedies.—DR. HENNING, *in Med. and Surg. Rep.*

**First Death from Bromide of Ethyl.**—Dr. J. Marion Sims reports in Medical Record, April 3d, a death from the use of bromide of ethyl, as an anesthetic. The operation in which it was used lasted an hour and a half. This is the first death from this drug we have observed reported. It is also the longest operation made under the influence of this anesthetic. The successful cases all occurred in short operations—not over forty minutes. Lesson from Dr. Sims: He caut ons about using it in long operations, or in cases suspected of having organic diseases of the kidneys. From the carefully elaborated report of the above-mentioned case, and from the discussion that has esulted therefrom, we are not convinced that the anesthetic was the prime cause of the death. Battey's operation very frequently results in the patient's death, even when performed by the best operators and under the most favorable circumstances. All of the subjects of this operation have for a long time suffered frequently repeated disturbances of the nervous system. So profound are the effects of these disturbances that recourse is had to this operation only to prevent epilepsy, insanity, or some other disorder that practically ruins all future prospects of happiness or healthful activity. In Dr. Sims' case the vicious circle had been maintained so long as to markedly impair all vital action of every organ in the body. We can understand that those organs should give way when called upon to endure the burden of an operation lasting for an hour and a half, and involving the most sensitive tissues in the body. There does not seem to be any reason to believe that a more fortunate result would have followed the use of ether or chloroform. Certainly from the same operation patients have died when the latter anesthetics were employed under circumstances as near like this one as we are likely to find. Doubtless the report alluded to will render surgeons more careful in the employment of this new anesthetic, a so render an important service to humanity.—*Det. Lancet.*

**Ozœna.**—The crusts which form in the nares in these cases should never be removed violently, as, in case hemorrhage be excited, they will always form again. They should be removed by post-nasal washings, or vapor or spray inhalations, and measures be taken to prevent their formation anew. Of these the best is the following ointment:

℞ Iodoform.................................. grs. v–viij.

Ether.......................................... fl. ʒj–fl. ʒjss.

Solve and add:

Vaseline...................................... ℥j.

Attar rosæ.................................. mv–viij.

Three measures are particularly condemned:

1. Plugging the nostrils.

2. Employing pure glycerine, because of its powerful attraction for water, which only serves to increase the dryness of the parts.

3. The use of alum, tannin, or any other astringent, for the same reason. Also the use of nasal snuffs.

Applications of calomel powder to the ulcerated surfaces are also condemned, and, instead, is recommended the internal administration of the iodides, local applications of solid nitrate of silver to the pharingeal ulcerations, post-nasal douches, and applications of solutions of sulphate of copper to nasal ulcerations, and constant inunction of the nostrils with the vaseline ointment. Nitrate of silver the author never uses in any form of throat disease, except syphilitic ulcerations, and then applied in the solid form to the exact spot. Even in these cases, the galvano-cautery, acid nitrate of mercury, or sulphate of copper is better. For disinfectants, the salycilates, thymol, and sanitas are preferred. General treatment directed to the particular diathesis of the patient is all important.—*N. Y. Med. Journal.*

**Treatment of Syphilitic Ulcerations by Pyrogallic Acid.** Vidal (Soc. de Therapeutique, Bull. Gen. de Therap., 1880, p. 184) has begun a series of researches upon pyrogallic acid, or pyrogallol. Pyrogallic acid, $C6\,H6\,O3$, obtained by the dry distillation of gallic acid heated to 200°-215° C., occurs in the form of needles or fine white scales. It melts at 115° C., and boils at 210° C. At 250° C. it decomposes into metagallic acid and water. It is soluble in two and one-half parts of water, and is also very soluble in alcohol, ether and glycerine. The aqueous solution becomes black when exposed to the air. It is neutral. M. Vidal made his first experiments with pyrogallic acid in 1878, employing it in the treatment of psoriasis. In Vienna it is used as a succedaneum to chrysophanic acid. It must be employed with caution, on account of its action upon the kidneys.

The syphilitic ulcerations in which M. Vidal employed pyrogallic acid were of a phagedenic character, and had resisted all previous efforts to stop their course. The acid was employed in the form of ointment, one part to five of lard, and was applied on three successive days; the pain caused was moderate, and only lasted eight or ten minutes. Under the action of these three cauterizations the surface of the phagedenic ulcers was decidedly modified. Three more applications were made, resulting in a rapid cure.

Since the first successful case M. Vidal employed this ointment for the purpose of dressing chancres, with very favorable results. The pure acid does not seem to have the same effects. He thinks it possesses an active affinity for neoplastic tissues. Dr. Dujardid-Beaumentz, however, does not agree with him on this point.—*Med. Times.*

**Influence of Singing upon Health.**—Prof. Manassein, of St. Petersburg, has published the result of a most interesting inquiry in which he has been engaged. He has made an examination of two hundred and twenty-two singers, varying in age from nine to fifty-three years, with particular reference to their chest measurement and pulmonary condition, as compared with persons who are not singers. He finds that the size of the chest is greater among singers than other classes, and that the relative advantage increases with the age of the vocalists.

Among singers who drink, he is convinced the habit prevents the due development of the chest. The vital capacity of the lungs is also greater in vocalists than in other persons.

Singers often suffer from soreness of the top of the throat, but seldom from bronchial catarrh; and the mortality from phthisis among them is, therefore, small. Vocalists, both drinkers and non drinkers, are, however, more subject to Bright's disease than people who do not sing. Singing is an excellent prophylactic for incipient consumption; and as a means for the development and strengthening of the chest is more efficacious than gymnastic exercises.—*N. Y. Med. Journal.*

